# Acute Caffeine and Coconut Oil Intake, Isolated or Combined, Does Not Improve Running Times of Recreational Runners: A Randomized, Placebo-Controlled and Crossover Study

**DOI:** 10.3390/nu11071661

**Published:** 2019-07-20

**Authors:** Gabrielle de Lima Borba, Julianne Soares de Freitas Batista, Ludmilla Marques Queiroz Novais, Myrnzzia Beatriz Silva, João Batista da Silva Júnior, Paulo Gentil, Ana Clara Baretto Marini, Bruna Melo Giglio, Gustavo Duarte Pimentel

**Affiliations:** 1 Laboratory of Research in Clinical Nutrition and Sports (Labince), Faculty of Nutrition, Federal University of Goiás, Rua 227, Quadra 68 s/n°, Setor Leste Universitário, Goiânia 74605080, GO, Brazil; 2 College of Physical Education and Dance, Federal University of Goiás, Goiânia 74605080, GO, Brazil

**Keywords:** coffee, caffeine, coconut oil, nutrients, running, performance

## Abstract

The aim was to evaluate the effect of caffeine (CAF) and extra virgin coconut oil (CO), isolated or combined, on running performance in runners. Methods: A randomized, placebo-controlled, and crossover study was conducted with thirteen recreational runners aged 18–40. All volunteers performed a 1600 m time trial at a 400 m track, each ingesting four different substances: (1) placebo (water), (2) decaffeinated coffee plus isolated CAF (DECAF + CAF), (3) decaffeinated coffee plus isolated CAF plus soy oil (DECAF + CAF + SO), and (4) decaffeinated coffee plus isolated CAF plus extra virgin coconut oil (DECAF + CAF + CO). The substances were ingested 60 min before the trials, the order of the situations was randomized, and there were one-week intervals between them. At the end of the trials, the Borg scale was applied to evaluate the rating of perceived exertion (RPE) and the time was measured. Results: Our data did not show differences in running time among the trials (placebo: 7.64 ± 0.80, DECAF + CAF: 7.61 ± 1.02, DECAF + CAF + SO: 7.66 ± 0.89, and DECAF + CAF + CO: 7.58 ± 0.74 min; *p* = 0.93), nor RPE (placebo: 6.15 ± 2.03, DECAF + CAF: 6.00 ± 2.27, DECAF + CAF + SO: 6.54 ± 2.73, and DECAF + CAF + CO: 6.00 ± 2.45 score; *p* = 0.99). Lactate concentrations (placebo: 6.23 ± 2.72, DECAF + CAF: 4.43 ± 3.77, DECAF + CAF + SO: 5.29 ± 3.77, and DECAF + CAF + CO: 6.17 ± 4.18 mmol/L; *p* = 0.55) also was not modified. **Conclusion**: Our study shows that ingestion of decaffeinated coffee with the addition of isolated CAF and extra virgin CO, either isolated or combined, does not improve 1600 m running times, nor influence RPE and lactate concentrations in recreational runners. Thus, combination of coffee with CO as a pre-workout supplement seems to be unsubstantiated for a short-distance race.

## 1. Introduction

Dietary supplements are widely used with the purpose of improving physical performance and preventing fatigue [1]. Among the supplements, caffeine (CAF, 1,3,7-trimethylxanthine) is one of the most consumed ergogenics in the world, and can be found in many foods and beverages, such as chocolate, teas, guarana, and coffee [2]. CAF has been studied because of its apparent positive effects in endurance sports [3,4,5]. During exercise, CAF can reduce the use of the glycogen and increase the release of free fatty acids [6,7], which might delay fatigue and increase endurance. The ergogenic potential of CAF might also be observed in the cellular matrix, where it acts as a competitive antagonist against adenosine receptors, releasing calcium to skeletal muscle, which is able to maximize the strength for muscular contractions [8,9].

Coconut oil (CO), in turn, is a saturated fat composed of about 50% medium chain fatty acids or medium chain triglycerides (MCT), namely lauric acid (C 12:0) and caprylic acid (C 8:0) [10]. However, less than 30% of lauric acid is released to the liver to be used as an energy source [11]. Fatty acids provide rapid energy availability and contain approximately 8–9 kcal per gram; however, because its structure contains many carbon atoms attached to oxygen, there is greater difficulty in oxidization [12]. Although no evidence demonstrated that pre-workout supplementation of long chain and medium chain fatty acids, as well as conjugated linoleic acid, has any effect on endurance performance [13,14], a recent study showed that four weeks (30 mL/d) of virgin CO associated with a single bout of moderate-intensity cycling in young adults was able to increase popliteal artery endothelial-dependent dilation, but did not change the post exercise-mediated hyperemia, nor plasma total antioxidant capacity [15]. Additionally, an isoenergetic ketogenic diet containing CO did not affect the run-to-exhaustion at 70% VO2max in men [16]. Although CAF is reported to positively effect physical performance [3,8], it is not fully understood whether or not CAF combined with extra virgin CO improves running time in humans. Likewise, it is unknown if this combination leads to an ergogenic effect.

Therefore, knowing the potential ergogenic effect of CAF, and that the pre-workout mix of CAF with extra virgin CO has been used in clinical practice without scientific evidence, we hypothesized that ingestion of decaffeinated coffee (DECAF) with CAF, but not with CO could improve running time. Thus, this study aimed to evaluate the effect of CAF and extra virgin CO, isolated or combined, on the running time in recreational runners.

## 2. Methods

### 2.1. Subjects and Recruitment

**Figure 1:**
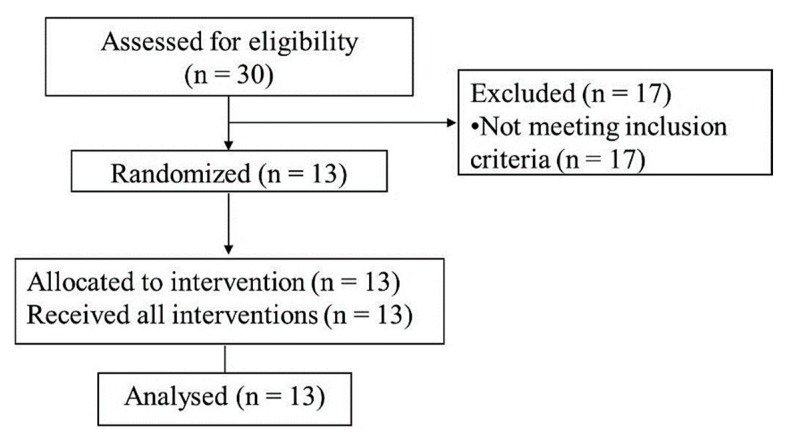
Flow diagram of study.

Thirty healthy recreational runners aged 18 to 40 initially volunteered to participate in the study. The volunteers were invited through social medial and by word of mouth. The exclusion criteria involved those who ran less than two times per week; having renal, cardiovascular, or hepatic diseases; being pregnant; having dietary restrictions; or currently using dietary supplements or anti-inflammatory medications. After removing a number of candidates based on the exclusion criteria, thirteen (8M/5F) volunteers were selected (Figure 1). All the runners signed a written consent that was approved by the Research Ethics Committee of the Federal University of Goias under the number 010883/2018.

After signing a written consent, the volunteers replied the initial anamneses one week before the familiarization test. This anamnesis consisted of body composition assessment, frequency of CAF consumption and habitual food intake evaluation.

### 2.2. Study Design

A randomized, placebo-controlled, and crossover design was conducted at the same time each day (~7 to 9 a.m.), to avoid possible circadian interferences. The study was conducted over a five-week period. In the first week, the volunteers did a five-minute warm-up and, after resting for 3–5 min, performed practice laps of 1600 m on the 400 m circuit where the time trial test would later take place to familiarize themselves with the track. On the day of the practice laps, the volunteers were instructed to run in the shortest possible time and the runner’s order was randomized using https://www.randomizer.org. During the second to fifth weeks, the trials were performed with the ingestion of different pre-test substances.

The volunteers were instructed not to do vigorous physical activities for a period of 24 h, and not to consume foods and beverages containing CAF in their composition (such as coffee, chocolate, maté, guaraná powder and soft drinks) and alcohol in the 48 h preceding the trials. In addition, they were strongly encouraged to maintain the same dietary and physical activity habits in order to avoid possible discrepancies in energy balance.

The tests were conducted in a randomized, crossover and placebo-controlled manner on the weekend (Saturday or Sunday).

The experimental tests consisted of four groups: (1) placebo: who receive 100 mL of warm water; (2) DECAF + CAF: decaffeinated coffee with 100 mL of warm water plus 6 mg/kg of CAF isolated; (3) DECAF + CAF + SO: decaffeinated coffee with 100 mL of warm water plus 6 mg/kg of CAF plus 15 g soy oil; and (4) DECAF + CAF + CO: decaffeinated coffee with 100 mL of warm water plus 6 mg/kg of CAF plus 15 g of extra virgin CO. All trials were separated by a one-week interval.

### 2.3. Supplementation

Administration of CAF anhydrous (6 mg/kg) was provided by the manipulation pharmacy and the amount was adjusted according to the weight of each runner [17,18]. The corresponding amount of CAF was weighed on an analytical balance (Shimadzu Automatic Digital Analytical Balance, ATX124; Kyoto, Japan) and kept in aluminium foil packets that were organized and identified by a qualified individual that was not participating in the research.

The extra virgin CO (Copra Alimentos, Maceió, Alagoas, Brazil, lot 792711818) and the refined SO (Leve, Imcopa^®^, Araucária, Paraná, lot c1017) were odorless and flavorless to minimize any bias, or identification, on the part of the participants in the study. SO was used because it presents improved palatability, similar to that of CO [10]. The amount of both oils of 15 g is equivalent to 16.5 mL. At the time of the supplement manipulation, 20 mL syringes were used to better determine the amount of either CO or SO.

Supplements were administered in unmarked containers and handled by a qualified individual who was not involved in the research, so researchers and runners would not know which supplements would be given. The DECAF coffee (DECAF coffee with water) was prepared 30 min prior to being ingested and standardized to be served using the same amount of coffee grinds (36 g coffee grinds in ½ L of mineral water) kept at ~45–40 °C and stored in bottles until the moment of the tests. The hot water trial was used for the placebo group, as in previous studies [19,20]. All substances, either containing DECAF coffee and oil (experimental groups) or only warm water (placebo groups), were ingested 60 min before participants started their time trial. Additionally, volunteers were questioned about which supplement they believed to have received.

### 2.4. Evaluation of Food Sources of Caffeine and Dietary Intake

The frequency of consumption of food sources of CAF was obtained using a previously-published adapted questionnaire [21].

Dietary intake was obtained using the 24 h food recall applied throughout the trials. From the total of six food recalls recorded, two were performed during participant recruitment (anamnesis application), one in the first time trial test, one in last week of the time trial tests, and two others on weekdays (in contrast to the normal time trial tests, which were held on the weekend).

The 24 h food recall was applied by a trained nutritionist. We obtained data about serving sizes, frequency and daily total calorie, carbohydrate, protein, lipids and water intake. For food intake analysis, DietPro^®^ software (version 5.8, Viçosa, Minas Gerais, Brazil) was used. The volunteers were encouraged to maintain their habitual food consumption during the whole experimental period. 

### 2.5. Running Trials

On the day of the trials, the participants woke up after eight hours of rest and were instructed to ingest their habitual breakfast without caffeine-sourced foods. After one hour, the volunteers went to the race track.

After supplementation, volunteers were instructed to remain in a resting state for 50–55 min. Approximately 5 min prior to the tests, a warm-up consisting of stretches and light walking was done. Then, 60 min after supplementation, the 1600 m time trial test on the 400 m outdoor race track commenced. The volunteers were instructed to run in the shortest possible time and this was recorded (in minutes) using a stopwatch.

There were no differences (*p* > 0.05) in the climatic features during the four days of data collection (temperature on first day: 20.5, second: 19.71, third: 20.8, and fourth: 19.8 °C, and relative humidity on first day: 54.0, second: 56.3, third: 54.0, and fourth: 61.1), as well as no difference in humidity.

### 2.6. Anthropometry, Blood Lactate, and Rating of Perceived Effort (RPE) Assessment

The anthropometric evaluation consisted of body weight, height, body mass index, and waist circumference. Moreover, skinfolds of the thighs, pectorals, supra iliac, and triceps were measured for calculation of body fat percentage [22,23].

Blood was collected from the finger at baseline and immediately after the tests. Lactate concentrations were determined using a portable lactate analyzer (Accutrend Plus, Roche^®^, Mannheim, Germany). The rating of perceived effort (RPE) was taken immediately after the trials using the Borg scale [24].

### 2.7. Statistical Analyses

The sample size was determined using the G*Power^®^ software version 3.1.9.2. The priori analysis of variance (ANOVA) test was used for repeated measurements, with 5% alpha error, 95% beta, and effect power of 0.75, resulting in a minimum sample of 13 volunteers. Shapiro–Wilk test was performed to check the normality of the variables and values were expressed as means ± standard deviation. Paired *t*-test was used to compare the food intake. ANOVA two-way was used to compare the effects of time x intervention of blood lactate concentrations. ANOVA one-way was performed to compare the delta of time trial performance and RPE score. Effect sizes were calculated using Cohen’d following the scale for interpretation <0.50 (small); ≥0.50 to <0.80 (medium); ≥0.80 (large). SPSS version 21.0 and Prism version 5.0 were used to perform the statistical analyses and graphs, respectively. The level of significance was set at 5% (*p <* 0.05).

## 3. Results

The volunteers’ characteristics are described in Table 1. They were young, eutrophic, and with normal waist circumference and body fat percentage. There was no difference between the two first trials compared with the two last trials for calories, macronutrients, amino acids, and water consumption (*p* > 0.05) (Table 2).

CAF was sourced mainly from the following: caffeinated coffee (85%), black tea (54%), soft drinks (54%), and chocolate (46%), and no volunteers reported ingesting guaraná powder, nor CAF alone. The weekly consumption of food/beverage sources containing CAF included the following: CAF coffee (4.18 times) > black tea (2.66 times) > chocolate (2.60 times) > soft drink (2.14 times). Therefore, all of the participants are moderate CAF consumers.

**Figure 2:**
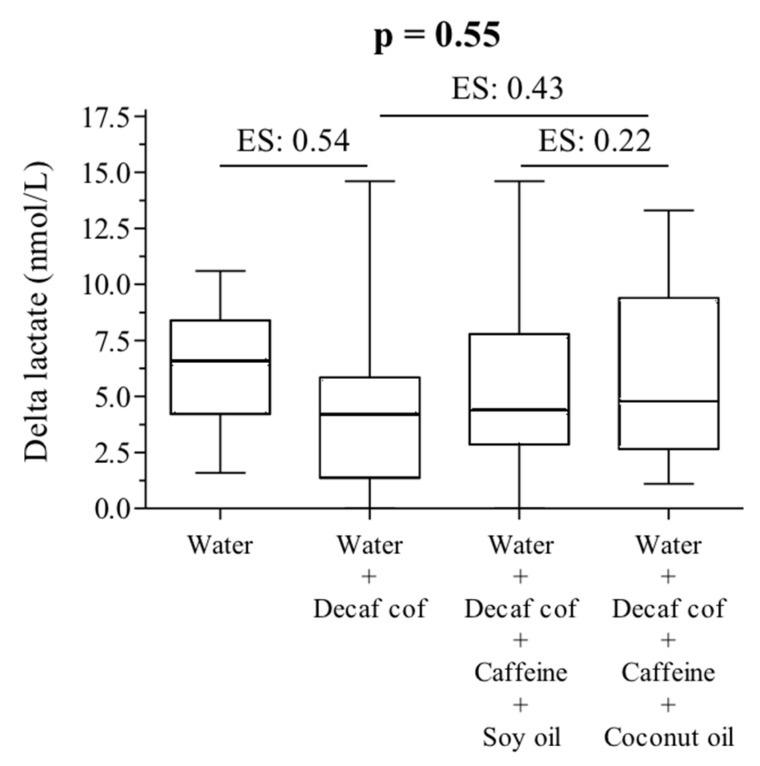
Delta of lactate concentrations. ES: effect size (small).

Blood lactate concentrations at pre- and post-test were not different between trials (placebo: 0.91 ± 0.36 to 7.14 ± 2.84, DECAF + CAF: 1.00 ± 0.50 to 5.43 ± 3.88, DECAF + CAF + SO: 0.95 ± 0.27 to 6.24 ± 3.86, and DECAF + CAF + CO: 1.12 ± 0.51 to 7.28 ± 4.00; *p* time < 0.001, *p* time × intervention *p* > 0.05), nor when evaluated using the delta values (placebo: 6.23 ± 2.72, DECAF + CAF: 4.43 ± 3.77, DECAF + CAF + SO: 5.29 ± 3.77, and DECAF + CAF + CO: 6.17 ± 4.18 nmol/L; *p* = 0.55, with small effect size between the groups) (Figure 2).

**Figure 3:**
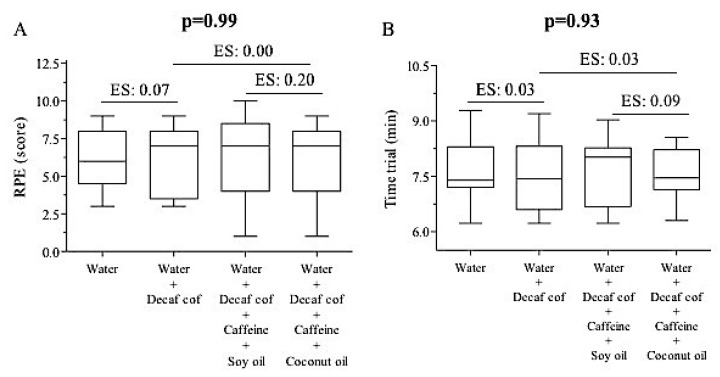
Rating of perceived exertion (RPE) (**A**) and time trial (**B**) performance at the end of running. ES: effect size (small).

RPE did not show any difference between the trials at the end of the trial (placebo: 6.15 ± 2.03, DECAF + CAF: 6.00 ± 2.27, DECAF + CAF + SO: 6.54 ± 2.73, and DECAF + CAF + CO: 6.00 ± 2.45 score; *p* = 0.99) (Figure 3A). No difference in running time was found (placebo: 7.64 ± 0.80, DECAF + CAF: 7.61 ± 1.02, DECAF + CAF + SO: 7.66 ± 0.89, and DECAF + CAF + CO: 7.58 ± 0.74 min; *p* = 0.93, with small effect size between the groups) (Figure 3B).

All volunteers correctly indicated the ingestion of placebo-water (*n* = 13), 92.3% DECAF + CAF (*n* = 12), 53.8% DECAF + CAF + SO (*n* = 7), and 46.1% DECAF + CAF + CO (*n* = 6) (*p* = 0.0004). Overall, correct identifications were made 73% of the time.

## 4. Discussion

To our knowledge, this is the first study to examine the ergogenic effects of pre-workout coffee ingestion combined with CO on running time in recreational runners. On the basis of our findings, a mixture of DECAF coffee with isolated CAF or extra virgin CO, either isolated or combined, does not improve 1600 m running times, nor change blood lactate concentrations and RPE.

Among the ergogenic properties of CAF is the increase in time trial performance, through mechanisms of central and peripheral action [25]. However, similar to the present study, two previous studies showed that DECAF coffee plus isolated CAF did not improve endurance performance [26,27], suggesting that other components of coffee can affect the ergogenic effect of CAF alone [27].

Contrary to our findings, previous studies showed that both CAF coffee (containing 5 mg/kg) or isolated CAF (5 mg/kg) is able to enhance endurance performance in cycling [28], and that CAF coffee improves 5 km time trial performance on the treadmill compared with DECAF coffee [29]. However, one of our previous studies found no difference in time trial performance in 800 m trials when comparing the effects of CAF coffee versus DECAF coffee [5]. Thus, one hypothesis for inconsistencies between studies may be related to how the CAF is administered, either isolated/alone or in a CAF coffee form.

In the present study, we did not find any difference in 1600 m performance between the consumption of placebo (water) compared with DECAF coffee plus isolated CAF. Similarly, a previous study also did not find any difference on endurance performance when comparing DECAF coffee versus the other three groups (either DECAF coffee plus isolated CAF or placebo) [27]. Although our data did not evaluate the blood CAF levels, Graham et al. [27] suggest that absence of an ergogenic effect may be independent of blood caffeine, paraxanthine, and theophylline concentrations, reinforcing the idea that the administration procedure (vehicle) appears to modify the ergogenic response of CAF. Likewise, other studies that combine DECAF coffee and isolated CAF or CAF coffee or isolated CAF alone require further investigation.

Church et al. 2015 [29] investigated the effects of CAF coffee or DECAF coffee ingested prior to a 5 km run on a treadmill, and no changes in blood lactate between trials were found. The findings of Church et al. 2015 [29] are in agreement with the present study, as we did not observe any differences in the blood lactate concentrations between groups. In addition, the lack of modification in lactate concentrations has already been found in previous work of our group [5], when testing the effects of CAF coffee compared with DECAF coffee on 1600 m running performance. Similar findings were reported by another group that also analyzed the effects of consumption of CAF coffee and isolated CAF alone on participants that ran to exhaustion [10].

Considering that high intensity exercise increased blood lactate concentrations [30] and the reduced consumption of fatty acids during exercise, we hypothesized that increases in lactate concentrations are time-dependent, but not those of fatty acids, as we used a short-term running protocol. In addition, pre-workout supplementation with long chain and medium chain fatty acids and conjugated linoleic acid does not produce an ergogenic effect [13,14]. Therefore, pre-exercise supplementation with fatty acids does not make sense in clinical practice.

Additionally, young adults that consume CO do not experience a change in total plasma antioxidant capacity following a bout of moderate-intensity cycling exercise [15] Likewise, it was found in rats that CO supplementation, with or without exercise, enhanced blood triacylglycerol and VLDL-c concentrations [31]. Therefore, lipid supplements did not seem to be a healthy option. Regarding performance, a recent study observed in men that a ketogenic diet containing CO did not change run-to-exhaustion with a 70% VO2max in men [16].

A previous study showed that theophylline, present in coffee, can also inhibit adenosine receptors and increase carbohydrate oxidation during a 30 min cycling exercise [32]. Thus, in the present study, theophylline may have influenced a greater availability of carbohydrates and lactate and, consequently, a greater energy supply in the trials, which may explain the non-effect of the additional energy supply from either CO or SO. However, the placebo group received only water and, when compared with the DECAF coffee group, no ergogenic effect was reported. Thus, we suggested that breakfast prior to races may have indicated that extra calories negatively impacted the running performance among the groups, despite CAF or CO.

In the present study, we observed that the type of oil, whether SO or CO, did not influence palatability, as the volunteers were not able to distinguish them. Additionally, the taste identification of the oils was minimized, as the CO is unscented and tasteless, triggering many volunteers to not be able to identify the oil type when mixed with the coffee.

One important finding in the present study was that most of the participants were able to correctly identify the substances they took. Previous studies suggested that the placebo effect might be an important mediator of CAF ergogenic effects [33,34]. Therefore, precise identification of placebo may have been associated with an absence of improvement in performance in some runners.

Although the CAF ergogenic effect (3 and 6 mg/kg) on 30.6 °C and 50% relative humidity led to performance loss [35], our study done in cooler environmental conditions (range 19.7–20.8 °C) did not affect the running time. Therefore, it is unlikely that climatic conditions led to dehydration and dropping performance in all trials. In addition, one of our previous studies found no difference in sweating rate between acute CAF and placebo supplementations [36].

Some limitations must be acknowledged. First, the small number of participants; therefore, the data should be interpreted with caution. Second, we are unable to measure the blood CAF and its metabolites concentrations; thus, we can not to confirm that all participants achieved similar amounts of CAF and its metabolites following the race. Third, although temperature and humidity were measured, wind speed was not; thus, we recognize that this could have hampered the running performance.

## 5. Conclusions

Our study shows that the consumption of DECAF coffee with CAF and CO, isolated or combined, does not improve 1600 m running time, influence RPE, nor lactate concentrations in recreational runners. Thus, combination of coffee with CO as a pre-workout supplement seems to be unsubstantiated for a short-distance race.

## Figures and Tables

**Table 1:** Characteristics of volunteers.

Variables	Mean ± SD
Age (years)	28.46 ± 5.63
Body mass index (kg/m^2^)	23.58 ± 3.90
Waist circumference (cm)	75.88 ± 11.24
Body fat (%)	16.19 ± 6.00

**Table 2:** Food intake among the volunteers.

Nutrients	Initial	Final	*p*
Calories (kcal)	2439.28 ± 948.33	2298.50 ± 672.15	0.666
Calories (kcal/kg)	35.48 ± 9.78	35.04 ± 10.90	0.915
Carbohydrate (%)	47.64 ± 7.77	51.59 ± 17.06	0.455
Carbohydrate (g)	282.29 ± 107.88	288.68 ± 100.64	0.877
Protein (%)	20.19 ± 3.69	20.67 ± 5.93	0.807
Protein (g)	120.64 ± 51.67	117.47 ± 50.43	0.876
Isoleucine (g)	3.02 ± 1.93	3.16 ± 2.04	0.862
Leucine (g)	5.41 ± 3.37	5.77 ± 3.74	0.797
Valine (g)	3.51 ± 2.28	3.57 ± 2.21	0.954
Lipids (%)	32.72 ± 5.81	33.06 ± 9.27	0.913
Lipids (g)	92.23 ± 48.02	82.15 ± 26.42	0.858
Water intake (L)	2.10 ± 1.01	2.06 ± 0.82	0.856

Values are expressed in means ± standard deviation.
